# The Ninth International Medical Congress

**Published:** 1886-09

**Authors:** 


### THE NINTH INTERNATIONAL MEDICAL CONGRESS.

"The *Lancet* mentions a number of well-known British Medical men, who entertain the intention of going to the Washington Congress, including, Mr. John Simon, Dr. B. W. Richardson, Dr. Thudichum, Sir James Paget, Sir Andrew Clark, Sir Spencer Wells, Professor John Chiene, Professor Fraser and Sir William Turner," and adds. "It is not America alone that is interested in the success of the meeting at Washington, but the profession throughout the whole world, and, we might add the world itself. When our profession meets, internationally, it is of good omen. We not only stimulate fraternal and scientific rivalry among ourselves, but every thought in advance and every medical discovery is a great boon for the human race and for all nations. We urge on members of our profession in the empire to strain a point to be at Washington, on or before September, 1887, where, if report is to be trusted, a very hospitable reception awaits them."—*New York Medical Journal.*

This list, as we readily see, contains the names of many distinguished British physicians and surgeons; doubtless many more equally as celebrated could be added to the list. From Germany, France, and, in fact, from the whole continent, come notices of this same tenor. Many, no doubt, will stay away on account of the unseemly controversy which has arisen concerning the organization of the congress. Without assuming to decide the question, we do most earnestly entreat our fellow pactitioners to forget the past and join in giving our brethren from abroad a hearty welcome, and to do everything in their power to make this congress a success and "a great boon for the human race and for all nations."

---

415 PEARL STREET, Buffalo, N. Y.

*Dear Doctor:*—I take this method of informing you that I make a specialty of Diseases of the "Genito-Urinary Organs," to which my practice is very largely limited.

Many years' study and practice in the large Philadelphia and

United States Marine Hospitals, as well as in private, has given me unusual opportunities for observation in this field.

My object in communicating this fact is to ask that you bear it in mind, and should any cases arise in your practice, which from their character, complications, or other reason you may not feel inclined to give your time and attention to that you will kindly refer them to me.

Yours very truly,

W. H. HEATH,

*Surgeon to the Sisters of Charity Hospital, Buffalo, N. Y.*

As will be seen from the above, Dr. Heath has had a large experience in this branch of medicine. Indeed the recognition by the profession in general of the Doctor's attainments in this direction is what, undoubtedly, has led him to make this step. It is unnecessary for us to add that he is well qualified to practice this branch as a specialty. Specialties, in this age, seem to be a necessity, both because superior knowledge is needed and because physicians practicing a specialty are willing to devote more time to complicated cases. We wish the Doctor success.

---

We are gratified to chronicle the full restoration to health of our late editorial confrere, Dr. Herman Mynter, who sailed for Denmark, on the 29th ult., to be absent several months. We learn that it is Dr. Mynter's intention to devote the autumn and winter to study and travel, and to return to our city the coming spring to engage in practice. We wish our esteemed brother, the success which he so richly deserves, and, on his return to his adopted city, the full restoration of his former large and lucrative practice. We can assure him that the medical profession of this city, with one voice, tender their best wishes for health and happiness, a hearty bon voyage and a safe return, with physical forces equipped for the active duties of the profession which he has so much adorned.

